# Evaluation of the Comparative Efficacy of Honey Thermal Microcautery, Standard Physiotherapy, and Sida cordifolia Oil via Nasal Administration in the Management of Frozen Shoulder: Protocol for a Randomized Controlled Trial

**DOI:** 10.2196/64066

**Published:** 2025-10-10

**Authors:** Shalini Pathania, Shweta Parwe, Punam Sawarkar, Milind Nisargandha

**Affiliations:** 1 Mahatama Gandhi Ayurved College, Hospital And Research Centre, Salod, Wardha, Maharashtra, 442001, India; 2 Sundarlal Patwa Government Medical College, Mandasaur, Madhya Pradesh, India

**Keywords:** frozen shoulder, range of motion, placebo, Sida cordifolia, physiotherapy, thermal microcautery

## Abstract

**Background:**

Frozen shoulder is an ailment that denotes dysfunction in the arm characterized by limited range of motion accompanied by pain. The prevalence of adhesive capsulitis is 3% to 5% in the general population and up to 20% in those with diabetes. Physiotherapy, analgesics, corticosteroids, and surgical capsulotomy are common forms of treatment. Administering oil through the nasal route (or *nasya karma*) is mentioned in the Ayurvedic scriptures for managing neck and clavicle disorders. Thermal microcautery (or *agni karma*) is a parasurgical procedure for treating related pathologies of bodily humors (*vata* and *kapha*). This study will aim to compare the efficacy of honey thermal microcautery, standard physiotherapy, and *Sida cordifolia* oil via nasal administration in the treatment of frozen shoulder to determine which provided the most relief.

**Objective:**

The primary aim is to evaluate the efficacy of honey thermal microcautery, standard physiotherapy, and *S cordifolia* oil via nasal administration using the visual analogue scale, range of motion, the Shoulder Pain and Disability Index, and the McGill Pain Questionnaire and compare these interventions. The secondary objective is to assess sustained relief in all 3 groups.

**Methods:**

We will enroll 60 patients, 20 in each group, for this single-blind assessor-controlled study. Group A will receive Ayurvedic treatment, that is, thermal microcautery using honey for 2 days (the first and seventh) and placebo capsules (twice per day) for 7 days, group B will receive standard physiotherapy and placebo capsules (twice per day) for 7 days, and group C will be given *S cordifolia* oil via nasal administration with 8 drops in each nostril and placebo capsules (twice per day) for 7 days. The evaluation parameters are pain (visual analogue scale), range of motion measured using a goniometer, the Shoulder Pain and Disability Index, and the McGill Pain Questionnaire. On September 18, 2023, approval was received from the Institutional Ethics Committee of Mahatma Gandhi Ayurved College Hospital and Research Centre (MGACHRC/IEC/Sep-2023/740).

**Results:**

The results will be subjected to statistical analysis using appropriate methods such as ANOVA. If the ANOVA shows significance, post hoc tests (eg, the Tukey honestly significant difference test) will identify group differences, with *P*<.05 considered significant. As of January 2025, a total of 16 patients have been recruited, divided into 3 groups, and the final results are expected to be published in November 2025.

**Conclusions:**

This comparative study seeks to establish the most effective treatment among honey thermal microcautery, standard physiotherapy, and *S cordifolia* oil *nasya* for managing frozen shoulder, potentially offering new integrative approaches to treating this condition.

## Introduction

### Background

Frozen shoulder (FS) is an ailment frequently seen in general practice. The prevalence of FS, also referred to as adhesive capsulitis and periarthritis, is between 3% and 5% in the general population and up to 20% in those with diabetes. It affects people aged between 40 and 60 years more frequently, including women and manual laborers [1,2]. In patients with diabetes, the incidence is 2 to 4 times higher than in the general population [3]. The prevalence of people who experience pain in the other shoulder after the first shoulder has healed is 6% to 17%, usually within 5 years. It is slightly more likely that the nondominant shoulder will be impacted [4]. The range of motion (ROM) in all directions is significantly reduced in FS (capsular pattern). It results from swelling, scarring, thickening, and shrinking of the capsule that typically encloses the shoulder joint [5]. Global fibroplasia results from a disruption in local collagen translation in FS, which is characterized by severe inflammatory changes in the capsule that point to the involvement of inflammatory mediators (interleukins, cytokines, B and T lymphocytes, growth factors, matrix metalloproteinases, tumor necrosis factors, and fibroblast activation markers) [6-9]. Synovitis-related inflammatory response in the capsule precedes the fibrotic rigidity of the capsule in cases of FS. Using nonsteroidal anti-inflammatory drugs, steroids, physiotherapy, and occasionally hydrodilatation and surgical procedures is the first line of treatment for FS. For most patients, conservative treatment, which includes corticosteroid infiltration and physiotherapy, is considered a suitable choice [1]. Still, despite conservative care, up to 50% of patients claim to have persistent pain, and up to 60% have noticeable motion restriction [10,11].

Steroid injections offer strong anti-inflammatory effects for FS, but potential complications such as pain and glucose changes may prevent patients from accepting this method. Patients endure lengthy rehabilitation times accompanied by a significant degree of pain and everyday disabilities [12,13]. Hence, an alternative Ayurvedic therapy is required in treating FS.

By using Ayurvedic principles, FS can be managed effectively [14] as several studies have investigated the efficacy of Ayurvedic treatments for FS [15]. There are few published clinical studies and case reports on the efficacy of Ayurveda interventions such as *laghumasha tail nasya* [16], *mahamasha taila nasya* [17], *agni karma* with *panchlohashalaka* [18], and *agni karma* with *tamra shalaka* [19].

One study was carried out at the Chaudhary Brahm Prakash Ayurved Charak Sansthan hospital on the efficacy of *mahamasha taila brihana nasya* and *agni karma* and found significant results [20].

### Rationale of the Study

The 3 arms in our study were selected considering their unique mechanisms of action to adequately treat FS. Honey thermal microcautery is an Ayurvedic treatment with thermal effects. Standard physiotherapy is the classic gold standard, ensuring a robust comparator. *Sida cordifolia* oil via nasal route was selected considering its efficacy for *vata-kapha* disorders, particularly musculoskeletal diseases. These treatments were selected considering the evaluation and comparison of diverse approaches between modern medicine and traditional medicine toward optimal outcomes.

No study has been published comparing honey thermal microcautery, standard physiotherapy, and *S cordifolia* oil nasal administration. Additionally, no study has explored the use of honey thermal microcautery in FS. Considering these gaps, this study is essential to determine the most effective and safe therapeutic approach for FS, evaluating interventions such as honey thermal microcautery, standard physiotherapy, and *S cordifolia* oil nasal administration to identify treatments that optimize recovery time and minimize associated concerns.

## Methods

### Overview

This study is an interventional, randomized, single-blind (assessor), 3–parallel arm study to compare the efficacy of honey thermal microcautery versus standard physiotherapy versus *S cordifolia* oil via nasal administration in the management of FS. We will intervene using honey thermal microcautery 2 times (the first and seventh day) along with placebo capsules twice daily for 7 days. Standard physiotherapy will be administered once a day along with placebo capsules twice daily for 7 days. *S cordifolia* oil via nasal administration will be administered via 8 drops in each nostril along with placebo capsules twice daily for 7 days. Follow-up will be conducted on the 8th, 16th, and 32nd days. Placebo capsules will be administered to reduce the psychological effect that patients might feel from knowing that they are not receiving any oral medicine, especially in the honey thermal microcautery group, where the treatment is administered only 2 times a week.

### Objectives

This study aims to determine the comparative effectiveness of honey thermal microcautery, standard physiotherapy, and *S cordifolia* oil nasal administration in managing FS. The primary objectives are to quantify pain reduction, measured via the visual analogue scale (VAS), and assess improvements in ROM, as well as administer the McGill Pain Questionnaire and Shoulder Pain and Disability Index (SPADI) for each treatment group at specific time points: 8 days, 16 days, and 32 days after the intervention.

### Secondary Objective

The secondary objective is to evaluate and compare the sustained relief on the 32nd day in each group, defined as maintaining at least a 50% improvement in the VAS score, ROM, McGill Pain Questionnaire score, and SPADI score compared to baseline measurements.

### Study Setting

The participants for this trial will be selected from specialized peripheral camps conducted in various urban and rural areas surrounding Wardha, Maharashtra, by the Datta Meghe Institute of Higher Education and Research, Mahatma Gandhi Ayurved College Hospital and Research Centre and the *panchakarma* (a prominent Ayurvedic tool) outpatient and inpatient department of Mahatma Gandhi Ayurved College Hospital and Research Centre.

Patients of either gender aged between 20 and 60 years clinically diagnosed as per the *International Statistical Classification of Diseases, 10th Revision* criteria code 75.00 regarding primary first- and second-grade FS will be selected.

The 2013 SPIRIT (Standard Protocol Items: Recommendations for Interventional Trials) guidelines will be followed for this study.

Simple randomization will be conducted using a computer-generated table. The participant enrollment process, allocation sequence creation, and intervention assignment will be handled by the researcher.

### Inclusion and Exclusion Criteria

This study will include (1) patients who are willing to provide written informed consent; (2) patients who are fit for nasal oil administration as per classic Ayurvedic guidelines [21] (ie, those who are free of indigestion; have not consumed food or alcohol recently; or do not have severe thirst, hunger, or exhaustion); (3) patients of either gender aged 20 to 60 years [22]; (4) patients with controlled type 2 diabetes mellitus (fasting blood sugar ≤140 mg/dL and postprandial blood sugar ≤180 mg/dL); and (5) patients with primary FS at the inflammatory (first) and frozen (second) stages. This study will exclude patients who are unfit for thermal cauterization [23]; patients who are contraindicated for physiotherapy (eg, those with epilepsy or a pacemaker); patients with trauma or injury (eg, dislocation or fracture of the shoulder and clavicle); patients with uncontrolled hypertension (160/100 mm Hg); patients who have rheumatoid arthritis, spinal canal stenosis, all types of malignancies, and stroke; patients with infectious conditions or systemic disorders; patients who are indicated for FS surgery; postoperative patients with FS; and pregnant and lactating women.

### Strategies to Improve Adherence to the Study Protocol

The participants will be assessed every day during the 7-day treatment period for procedure adherence and placebo capsule consumption. The participants will be asked to return the empty medicine container at the end of the study. Repeated phone reminders to regularly take the medicine from project staff will be given to participants or their family members.

### Intervention Description

This study involves 3 groups, each with specific treatment protocols, shown in Table 1. All 60 eligible participants will be thoroughly informed about the study protocol, and their consent will be obtained. They will be randomly divided into 3 groups: group A, group B, and group C. In group A, participants will receive honey thermal microcautery 2 times (first and seventh days) and placebo capsules for 7 days. Group B will receive standard physiotherapy once a day along with placebo capsules for 7 days. Group C will receive *S cordifolia* oil via 8 drops in each nostril and placebo capsules for 7 days. All baseline parameters will be recorded at the beginning of the trial. This study conforms to the SPIRIT guidelines, ensuring standard execution and ethical handling of the clinical trial. Placebo capsules will be identical in appearance, taste, and administration schedule to mimic a real medication. These will be administered alongside the active intervention twice daily after food, ensuring blinding and consistency throughout the study.

**Table 1.** Grouping and posology.

Group	Intervention	Dose and frequency	Procedure time (min)	Duration (d)	Follow-up
A	Honey thermal microcautery+placebo	2 times a wk	20-30	2 (first and seventh)	On the 8th, 16th, and 32nd days
B	Standard physiotherapy+placebo	Once a day (in the morning)	30-60	7	On the 8th, 16th, and 32nd days
C	*Sida cordifolia* oil via nasal route+placebo	8 drops once a day	20-30	7	On the 8th, 16th, and 32nd days

Study drugs will be stored in a secure location and administered to the participants only under the direction of the investigator. Drug dispensing records will be kept. Any imbalance in the quantities dispensed and returned will be explained.

### Properties of the Drugs Used in *S Cordifolia* Oil

The properties of the *S cordifolia* ingredients are shown in Table 2. The available *S cordifolia* oil of market preparation (*sahastra yoga*) [24] will be used. The treatment group medicine will be procured from good manufacturing practice–certified pharmacies to ensure the product’s safety and quality standards.

**Table 2.** The names of the drugs used to prepare *Sida cordifolia* oil with their Latin names and properties and the parts used.

No.	Ingredient	Botanical name	*Rasa* ^a^	*Guna* ^b^	*Virya* ^c^	*Vipaka* ^d^	*Doshaghnata* ^e^	Part used
1	Bala^g^	*S cordifolia*	Madhura^j^	Guru^l^, snigdha^m^, and pichhila^n^	Sheeta^o^	Madhura^j^	Vatapittashamak^s^	Root
2	Tila taila^h^	*Sesamum indicum*	Madhura^j^ and tikta^k^	Guru^l^	Ushna^r^	Ushna^r^	Vatakaphahara^t^	—^f^
3	Godugdha^i^	—	Madhura^j^	Sheet^o^, snigdha^m^, Kinchidkledkarka^p^, and kinchidguru^q^	Sheet^o^	Madhura^j^	Vatpittashamak^s^	—

a Taste.

b Qualities.

c Potency.

d Postdigestive effect.

e Postdigestive action on doshas.

f Not used.

g Country mallow

h Sesame oil

i Cow milk

j Sweet

k Bitter

l Heavy

m Unctuous

n Slimy

o Cold

p Mildly unctuous

q Mildly heavy

r Hot

s Pacifies both Vata and Pitta (bodily humor)

t Removes aggravated Vata and Pitta

### Procedure for Oil Administration Through the Nostrils

Oil administration through the nostrils is conducted in 3 phases [25]: preprocedure (*poorvakarma*), main procedure (*pradhanakarma*), and postprocedure (*aschatkarma*).

#### Preprocedure (*Poorvakarma*)

##### Collection of Material (*Sambar Sangraha*)

The procedure will be conducted in a room free of dust and direct flow of air; with appropriate lighting; and equipped with a table, a chair, medicine for nasal administration, an instrument for administering oil through the nasal route, a towel, cotton, a smoke-producing instrument, a spitting vessel, oil for massage, and material for sudation.

##### Examination of the Patient

Patients unfit for nasal oil administration will be excluded. Informed consent will be obtained, and the patients will be instructed to brush their teeth and clean their mouths.

##### Positioning

The patient will recline comfortably, either sitting with the neck slightly tilted backward or lying on a table with the head supported by a towel. A gentle massage with sesame oil will be applied to the face and head, followed by mild face heating.

### Main Procedure (*Pradhana Karma*)

The patient’s eyes will be covered with a cloth. Eight drops of *S cordifolia* oil will be gradually administered one by one into the distal portion of both nostrils using the index finger of that practitioner’s hand and cotton.

A gentle massage will be administered to the forehead, cheeks, hands, and feet. The patient will be instructed to spit out any excess oil that reaches the mouth. The patient will be kept lying down while watching any symptoms that develop.

### Postprocedure (*Paschatkarma*)

The patient will rinse their mouth with warm water and gargle. Mild medicated *Curcuma longa* smoke will be inhaled to clear the throat. The patient will rest in a warm, wind-free environment; follow a light diet; and drink warm water.

### Thermal Microcauterization Procedure

#### Preprocedure (*Poorva Karma*)

The patient will be informed about the procedure, and written consent will be obtained. A pinpointed metallic rod will be made red hot by directly heating it on a fire and smeared with honey [26]. The affected shoulder will be cleaned using a povidone solution, and tender points will be marked.

#### Main Procedure (*Pradhan Karma*)

The heated rod will be applied to the marked tender points by keeping a 0.5- to 1-cm distance for approximately 1 second at each end. The number of burns will be determined based on the patient’s tolerance and the number of tender points.

#### Postprocedure (*Paschat Karma*)

Aloe vera pulp will be applied to the treated areas, and *Glycyrrhiza glabra* powder will be sprinkled. The patient will be advised to avoid trauma, exertion, and water on the treated site for at least 12 hours.

### Standard Physiotherapy Procedure

#### Preprocedure

The patient will be informed about the exercises, and written consent will be obtained. The patient will be instructed on proper technique and complete preparatory exercises (pendulum swing) [27].

#### Main Procedure

For the shoulder wheel, the patient will stand sideways, align their shoulders with the wheel’s axis, and perform 10 anterior and 10 posterior rotations. Abduction and flexion exercises on a wall-mounted shoulder finger ladder will be conducted next. Patients will stand sideways facing the ladder and perform each movement for 10 repetitions.

#### Postprocedure

The patient will rest for 5 to 10 minutes.

### Outcomes

This study aims to evaluate and compare the efficacy of honey thermal microcautery, standard physiotherapy, and *S cordifolia* oil vial nasal administration using criteria such as the VAS, ROM, the McGill Pain Questionnaire, and the SPADI in patients diagnosed with FS.

#### *Ruja* (Pain; VAS)

The patient will be asked to rate the intensity of the pain they are experiencing on a scale from 0 (no pain) to 10 (worst pain) [28].

There will be 5 pain subcategories: no pain, mild pain, moderate pain, severe pain, and intolerable pain. Numbers from the scale will be used to measure the intensity of the pain, as shown in Table 3. A score of 0 will be considered no pain, a score of 1 to 3 will be considered mild pain, a score of 4 to 6 will be considered moderate pain, a score of 7 to 8 will be considered severe pain, and a score of 9 to 10 will be considered intolerable pain. Identifying patients with FS using this VAS pain assessment will make it easier to include them in this study for treatment.

**Table 3.** The grading on the visual analogue scale (VAS) for frozen shoulder.

Pain level	Pain grade (VAS score)
No pain	0
Mild pain	1 (up to 3)
Moderate pain	2 (4-6)
Severe pain	3 (7-8)
Intolerable pain	4 (9-10)

#### Normal ROM of the Shoulder Joint

The goniometer measures the shoulder’s ROM before and after treatment [29].

Flexion, extension, abduction, adduction, and internal and external rotation are possible shoulder movements, and the normal readings for these movements are shown in Table 4.

**Table 4.** The regular movements and readings for the shoulder joint.

Movement	Normal reading (degrees)
Flexion	180
Extension	50
Abduction	180
Adduction	50
Internal rotation	90
External rotation	90

All the ROM measurements will be taken in a standing position, and the participant will be instructed to place the back of the head, back and buttocks, and heels against the wall to prevent the trunk from moving. Each movement will be measured 3 times on a goniometer at the glenohumeral joint to avoid human error, and the average of 3 readings will be calculated and recorded.

#### SPADI Measurement

The pain scale and disability score will be decided based on a questionnaire administered by the researcher to the participants [30], as shown in Multimedia Appendix 1. Verbal anchors for the pain dimension are “no pain at all” and “worst pain imaginable,” and those for the functional activities are “no difficulty” and “so difficult that it required help.” The scores from both dimensions are averaged to derive a total score.

#### Interpretation of Scores

The total pain score as a percentage is calculated using the following formula: total obtained score / 50 × 100. If a participant does not answer all questions, the score is divided by the total possible score (eg, if 1 question is missed, the score is divided by 40).

The total disability score as a percentage is calculated using the following formula: total obtained score /80 × 100. If a participant does not answer all questions, the score is divided by the total possible score (eg, if 1 question is missed, the score is divided by 70).

The total SPADI score as a percentage is calculated using the following formula: total obtained score /130 × 100. If a person does not answer all questions, the score is divided by the total possible score (eg, if 1 question is missed, the score is divided by 120) The means of the 2 subscales are averaged to produce a total score ranging from 0 (best) to 100 (worst).

#### McGill Pain Questionnaire

The McGill Pain Questionnaire assesses pain through 3 components: sensory, affective, and evaluation dimensions [31]. Patients select descriptors that match their pain experience, and pain is related to intensity using a present pain intensity scale. Results are interpreted by summing the pain rating index.

### Participant Timeline With Deliverables

Table 5 outlines the study’s timeline, highlighting each phase’s duration and overlapping activities systematically. The total study period is 24 months, including preparatory work, recruitment, intervention, follow-up, and analysis and comprises 3 cycles (ie, 1-3.5 months for the drugs), including buying the drugs. Recruitment of the staff, procuring the equipment, and finalizing the case report form (CRF), among other related activities, were completed in the initial phase, not throughout the full 24 months. Participants will be recruited over 16 months, the treatment period will last 7 days, and the last follow-up will be on the 32nd day. Data compilation, analysis, report preparation, and publication will be conducted during the last 3 months.

**Table 5.** Gantt chart.

Step	Quarter							
	1	2	3	4	5	6	7	8
Approval from IEC^a^	✓							
Review of the literature		✓						
Drug preparation			✓					
Enrollment of patients				✓	✓			
Data collection				✓	✓			
Statistical analysis				✓	✓			
Thesis writing						✓		
Submission						✓		

a IEC: institutional ethics committee.

### Sample Size

#### Formula Using Mean Difference

The sample size was calculated based on pilot study data due to the absence of larger studies on this topic as follows:

$n_{1}=n_{2}=2\cdot \frac{(Z_{\alpha }+Z_{\beta }{)}^{2}\sigma^{2}}{(\delta {)}^{2}}\;\begin{array}{ll} Z_{\alpha }=1.96 & \text{at 5\textbackslash{}\% error \textbackslash{}§amp; CI at 95\textbackslash{}\%}\\ Z_{\beta }=0.84 & \text{Power at 80\textbackslash{}\%} \end{array}$

#### Primary Variable: Abduction Improvement

The primary variable, shoulder abduction improvement, was used to calculate the minimum sample size required for the study, ensuring adequate power to detect a meaningful difference between groups. The primary variable in this study is shoulder abduction improvement, measured as the increase in the range of motion after the intervention. Based on expected differences, variability, significance level, and power, the minimum sample size required was calculated to ensure reliable detection of a meaningful improvement

Where the mean score for abduction in *shudhabala taila nasya* pre=2.23 (as per the study by Revathi et al [22]).

Mom score for abduction in *shudhabala taila nasya* post at follow-up=1.06.

Difference (pretest-posttest)=1.17

SD=0.58.

Considering 45% superiority in the thermal microcautery group=(1.17) × 45 = 0.526

Mean differenсе *δ*=0.526

#### Minimum Sample Size Required

Based on expected differences, variability, significance level, and power, the minimum sample size required was calculated to ensure reliable detection of a meaningful improvement:

n=1=2=2 × (1.96 + 0.84)^2^ × (0.58)^2^/(0.526)^2^=20 per group

Total samples = 20 × 3 per group=60

### Randomization and Allocation

Computer-generated random numbers will be used to create a randomization chart. The participants will be randomized at a ratio of 1:1:1. To guarantee that treatment assignments are balanced, allocation sequence creation will be conducted using permuted blocks and provided in a randomized table. The researcher will enroll and give the participants an intervention and allocation sequence.

### Study Procedure

#### Overview

Before any activities related to the trial are initiated, participants or their guardians will be provided with verbal and printed information about the study. The investigator will ensure that they fully understand the study’s objectives, procedures, possible discomforts, and expected benefits. Participation shall be voluntary, and participants shall retain their right to withdraw from the trial at any time without prejudice.

**Figure 1.**
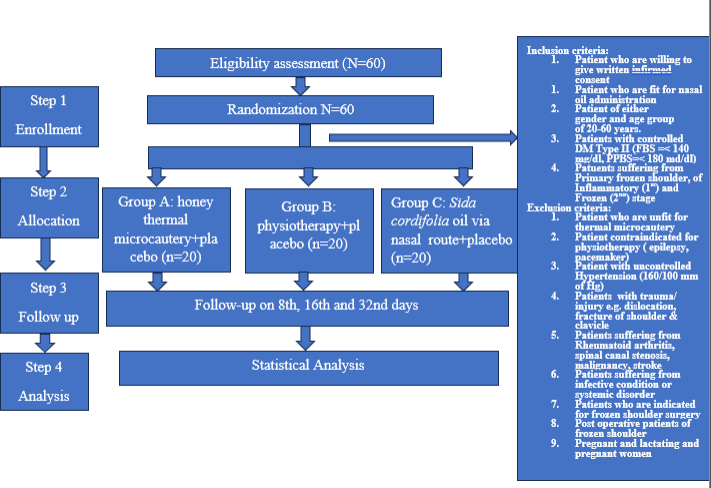
Study procedure.

After screening, suitable patients will be included in the study after signing the consent form. A required examination will be conducted. If the patients fall within the inclusion criteria, they will be included in the study with that day written down as day 1 or the baseline visit. They will be administered the treatment after completing all the formalities according to the protocol. Participants will visit the study site on procedure days and follow-up days during the trial. The activity details during each visit are shown in Table 6 and Figure 1.

**Table 6.** Study schedule.

Component	Screening (day X)^a^	Baseline (day 0)	Day 1	Days 2-6	Day 7	Day 8	Day 16	Day 32
Informed consent	✓							
Demographic information and medical history		✓						
Assessment of *prakriti^b^*		✓						
Clinical examination	✓	✓	✓	✓	✓	✓	✓	✓
Honey thermal microcautery			✓		✓			
Standard physiotherapy			✓	✓	✓			
*Sida cordifolia* oil administration via nasal route			✓	✓	✓			
Placebo capsule issue			✓					
Assessment of adverse reactions			✓	✓	✓	✓		
Follow-up visit						✓	✓	✓

a Day X refers to the variable screening day when a participant is evaluated before the official baseline (day 0) of the study.

b Body constitution

#### Withdrawal Criteria

The participants may be withdrawn from the trial under any of the following circumstances: (1) any adverse event or adverse reaction (AR), (2) voluntary withdrawal by the participant, (3) violation of inclusion or exclusion criteria, or (4) noncompliance with the treatment regimen (a minimum of 80% compliance is required to continue participating in the study).

### Data Collection Plan and Management

All the details entered in CRFs will be collected, and each participant will be provided with a unique enrollment number. The signed consent forms, along with the CRFs, will be placed in locked cabinets, and all electronic data will be password protected. Data access will be limited to the principal investigator and coinvestigator during and after the study. For dropouts, 10% of the data will be imputed using proper techniques, and efforts will be made to collect data for at least 1 month after enrollment.

Emergency unblinding will be conducted only if necessary for the participant’s safety or clinical management. If needed, the investigator will follow the emergency unblinding procedure. The allocation will not be revealed to the participant or study personnel unless necessary for patient care. All unblinding events will be recorded in the CRF.

### Statistical Methods

One-way ANOVA will be applied to compare the means of the 3 groups. If ANOVA indicates a significant difference, post hoc tests will be further applied (eg, the Tukey honestly significant difference test) to identify which group differs. A *P* value of <.05 will be considered statistically significant. Covariates such as age and baseline severity will be considered, and adjustments will be made using analysis of covariance if needed. The analysis will be intention to treat, with missing data handled through multiple imputation. Statistical assistance will be provided by a statistician or someone skilled in statistics. We will use SPSS (version 26; IBM Corp) for statistical analysis.

### Ethical Considerations

All study interventions and treatments will be provided completely free of cost, and participants will not be charged for any procedures, assessments, or related expenses.

#### Overview

Ethics approval from the Institutional Ethics Committee of Mahatma Gandhi Ayurved College Hospital and Research Centre was received (MGACHRC/1EC/Sep-2023/74) on September 18, 2023. The Clinical Trials Registry India registration number for this study is CTRI/2023/11/059594. The committee will oversee the trial’s advancement and determine its conclusion. The researcher shall evaluate any unfavorable event and report it to the ethics committee. The participants will be provided with written research details in 2 languages (Marathi and English), and written consent will be obtained from the participants on an informed consent form. They will be informed about the confidentiality of their details. Safety will be monitored by recording any adverse events during the entire study. We will make sure that no photos include any identifiable participants.

#### Consent

The patients will be fully informed about the study’s purpose and provide signed consent before the study commences. The researcher will personally ensure that participants understand the information and voluntarily agree to participate in the trial.

Private information about the participants will be collected and kept confidential before, during, and after the trial. The consent form will include specific information about the study purpose, procedures, potential risks and benefits, confidentiality measures, and the voluntary nature of participation. Participant comprehension will be assessed through a follow-up discussion, allowing them to ask questions and clarify their understanding.

#### AR Management

In the event of ARs during the trial, the affected participants will receive treatment at the institute’s attached hospital. The investigator will cover the costs associated with the treatment to ensure the participants’ safety.

#### Intervention Modification

Any negative effects noted during the treatment will be recorded and immediately reported to the ethics committee. The patients will receive the necessary treatment for the negative effects. If participants opt out of the treatment, they will be asked why, and this will be carefully documented.

### Previous and Concomitant Medication

Patients will be allowed to continue their allopathic medications for diabetes mellitus and hypertension, anticoagulant therapy, or hormonal medications as per their existing treatment regimen. Any analgesics will be discontinued during the study period to prevent bias or interference with the study outcome. The investigator will record any medication taken by the patients other than the protocol drugs via any route.

## Results

Preliminary data collection is ongoing, and participant recruitment for the study commenced in October 2024. As of January 2025, a total of 16 participants have been recruited across all 3 study groups. Data analysis is currently in progress. The final results of the study are expected to be published in November 2025. The study’s findings will be disseminated through peer-reviewed publications.

## Discussion

### Anticipated Findings

A group of 20 participants will receive honey thermal microcautery, another group of 20 participants will receive standard physiotherapy, and another group of 20 participants will receive *S cordifolia* oil through the nasal route. All 3 groups will receive the treatment for 7 days. On the 8th, 16th, and 32nd days, we will assess the patients using the VAS, the SPADI, the McGill Pain Questionnaire, and ROM. We will compare the changes before and after therapy. We expect that the interventional group, specifically the honey thermal microcautery group, will exhibit statistically significant outcomes.

The study evaluated the efficacy of *mahamasha taila brihana nasya*, administered as 8 drops in each nostril per session, over 2 sessions spaced 7 days apart, along with 3 sittings of *agni karma* performed once weekly on the same day. In contrast, the third group (group C), both *mahamasha tailabrihana nasya* and *agnikarma* were administered. The assessments were taken on days 0, 7, 14, 21, and 28 using the VAS, the SPADI, the McGill Pain Questionnaire, and ROM goniometric examination. Improvement was observed in all 3 groups, especially the group in which both treatment modalities were administered.

A systematic review [32] compiled different studies on FS and thermal microcautery.

Van’t Hoff’s principle states that, for every degree that the body temperature rises, the body’s basal metabolism increases by a specific proportion. An increase in temperature causes muscles to relax, which reduces discomfort and inflammation associated with muscle spasms. This can be achieved via thermal microcautery [33]. Similar to infrared fomentation, the light waves cause inflammation to be reduced by penetrating deeper into the body; thermal microcautery similarly causes the local capillaries to dilate, improving the blood supply to the affected area and eliminating inflammatory compounds [34].

Physiotherapy, such as wheel and step ladder exercises, aids in FS by enhancing joint mobility and flexibility. Wheel exercise allows for gradual stretching, whereas step ladder exercise improves shoulder strength and ROM. Together, they facilitate functional recovery and improve ROM. Our hypothesis is that honey thermal microcautery will yield more effective results than standard physiotherapy and *S cordifolia* oil via the nasal route because of its instant and satisfactory benefits. This research may guide future studies, incorporating traditional and modern methods, thus providing a well-rounded treatment for FS. This study seeks to guide clinical decisions by systematically comparing these interventions, enabling the choice of a more effective and individualized treatment for FS. This study compares diverse interventions, uses multidimensional assessment tools, and integrates Ayurvedic principles with modern therapy. It provides a new perspective into honey thermal microcautery; thus, clinicians can fine-tune effective treatments for FS. The limitation of this study is the small sample size, which, while adequate for preliminary insights, may limit the generalizability of the findings. Future studies with larger cohorts and extended follow-up periods are recommended to validate and enhance the applicability of the results. The primary findings will establish that honey thermal microcautery, standard physiotherapy, and *S cordifolia* oil via nasal administration effectively manage FS, ultimately determining which provides the best outcome.

### Conclusions

This study aims to evaluate the efficacy of *S cordifolia* oil administered through the nasal route, honey thermal microcautery, and standard physiotherapy for treating FS. We highlight that these are just preliminary theories based on available data, even though we anticipate that regular physiotherapy and honey thermal microcautery will show notable benefits. We will draw final conclusions about the efficacy of each treatment once data collection and analysis are finished.

## Supplementary material

10.2196/64066 Multimedia Appendix 1 The grading on the SPADI scale.

## Abbreviations

AR: adverse reaction
CRF: case report form
FS: frozen shoulder
ROM: range of motion
SPADI: Shoulder Pain and Disability Index
SPIRIT: Standard Protocol Items: Recommendations for Interventional Trials
VAS: visual analogue scale
