# Supplementary material for: Evaluation of the Comparative Efficacy of Honey Thermal Microcautery, Standard Physiotherapy, and Sida cordifolia Oil via Nasal Administration in the Management of Frozen Shoulder: Protocol for a Randomized Controlled Trial
Source: JMIR Res Protoc. 2025 Oct 10;14:e64066. doi: 10.2196/64066 (PMC12513683; doi:10.2196/64066)
Supplement: Multimedia Appendix 1 [file resprot-v14-e64066-s001.docx]

**Name: Date:**

**SPADI (SHOULDER)**

**PAIN SCALE**

| **Question** | **Scale** |
| --- | --- |
| 1. At its worst. | No pain 0 1 2 3 4 5 6 7 8 9 10 Worst Pain Imaginable |
| 2. When lying on involved side. | No pain 0 1 2 3 4 5 6 7 8 9 10 Worst Pain Imaginable |
| 3. Reaching for something on a high shelf. | No pain 0 1 2 3 4 5 6 7 8 9 10 Worst Pain Imaginable |
| 4. Touching the back of your neck. | No pain 0 1 2 3 4 5 6 7 8 9 10 Worst Pain Imaginable |
| 5. Pushing with the involved arm. | No pain 0 1 2 3 4 5 6 7 8 9 10 Worst Pain Imaginable |

**DISABILITY SCALE**

| **Question** | **Scale** |
| --- | --- |
| 1. Washing your hair. | No difficulty 0 1 2 3 4 5 6 7 8 9 10 So difficult required help |
| 2. Washing your back. | No difficulty 0 1 2 3 4 5 6 7 8 9 10 So difficult required help |
| 3. Putting on an undershirt or pullover sweater. | No difficulty 0 1 2 3 4 5 6 7 8 9 10 So difficult required help |
| 4. Putting on a shirt that buttons down the front. | No difficulty 0 1 2 3 4 5 6 7 8 9 10 So difficult required help |
| 5. Putting on your pants. | No difficulty 0 1 2 3 4 5 6 7 8 9 10 So difficult required help |
| 6. Placing an object on a high shelf. | No difficulty 0 1 2 3 4 5 6 7 8 9 10 So difficult required help |
| 7. Carrying a heavy object of 10 pounds. | No difficulty 0 1 2 3 4 5 6 7 8 9 10 So difficult required help |
| 8. Removing something from your back pocket. | No difficulty 0 1 2 3 4 5 6 7 8 9 10 So difficult required help |
